# Infiltration in Pilocytic Astrocytoma: A Diagnostic Pitfall

**DOI:** 10.7759/cureus.27940

**Published:** 2022-08-12

**Authors:** Murad Alturkustani

**Affiliations:** 1 Department of Pathology, King Abdulaziz University, Jeddah, SAU

**Keywords:** pilocytic astrocytoma, infiltration, glioma, diffuse glioma, circumscribed, brain tumor

## Abstract

Glioma can be classified according to its infiltrative capacity into circumscribed and "diffuse"/infiltrative glioma. Pilocytic astrocytoma is typically grouped under the circumscribed astrocytic glioma in the 2021 World Health Organization (WHO) classification of central nervous system (CNS) tumors. The distinction of pilocytic astrocytoma from diffuse glioma is fundamental as it could be the difference between CNS WHO grade 1 and grade 4 glioma. This study aims to determine the infiltrative nature of pilocytic astrocytoma in different brain locations. All cases diagnosed as "pilocytic astrocytoma" were retrieved from 2008 to 2021. The clinical information (age, sex, location of the tumor), pathological description, and performed immunostaining were obtained from the pathological reports. The available pathological slides were retrieved and examined for the following features: diagnosis, infiltrative vs. circumscribed tumor, and immunostaining characteristics. There were 20 males and 19 females aged 17 months to 31 years. The diagnosis of pilocytic astrocytoma was confirmed in 38/39 cases, and in one case, the diagnosis changed to a dysembryoplastic neuroepithelial tumor. Histological infiltration is defined as the presence of neoplastic cells among the nonneoplastic brain parenchyma. Twenty cases were well-circumscribed with no evidence of infiltration histologically, while 18/38 cases showed apparent infiltration into adjacent brain tissue. The infiltration was not restricted to cerebellar pilocytic astrocytoma (12/24, 50%) but was also present in 3/7 supratentorial, single brainstem, and single spinal cord cases. In conclusion, cases with typical morphological features of pilocytic astrocytoma could show areas of brain infiltration, which should not affect the certainty of the diagnosis.

## Introduction

Pilocytic astrocytoma is a central nervous system (CNS) World Health Organization (WHO) grade 1 tumor that can affect the entire neuroaxis but with a predilection to the cerebellum and the optic pathway. It is the most common CNS glioma in children, and the incidence rate decreases with aging [1]. Pilocytic astrocytoma is grouped under the circumscribed astrocytic glioma in 2021 WHO CNS tumors [1]. However, early pathological literature described cases of “diffuse” cerebellar pilocytic astrocytoma [2] and pilocytic astrocytoma with infiltration [3]. Glioma can be classified according to its infiltrative capacity into adjacent brain parenchymal into circumscribed and diffuse/infiltrative glioma. In the diagnostic approach of an astrocytic tumor, the infiltrative growth can be used to favor the diagnosis of infiltrative glioma over pilocytic astrocytoma.

A general rule in the pathological grading of CNS glioma is that grading should be applied to the tumor after determining the morphological diagnosis. The significance of this rule is evident when dealing with a tumor where the differential is between pilocytic astrocytoma and infiltrative astrocytoma. The interpretation of the pathological features that are commonly used as “high-grade” pathological features (i.e., mitoses, necrosis, and microvascular proliferation) depends on the initial histological diagnosis of the tumor. While these features may be acceptable in CNS WHO grade 1 astrocytoma, their presence indicates a high-grade (grade 3-4) glioma in infiltrative astrocytoma. Similarly, the integration of the molecular features with the histological diagnosis depends on the initial diagnosis. While the presence of wild-type isocitrate dehydrogenase (IDH) is considered the classic feature in pilocytic astrocytoma, it may indicate CNS WHO grade 4 tumor in infiltrative astrocytoma [4]. Also, the presence of H3 K27M mutation in midline glioma indicates CNS WHO grade 4 glioma in an infiltrative tumor; its significance is unknown in pilocytic astrocytoma [5]. Therefore, the initial categorization of the glioma as pilocytic astrocytoma or as infiltrative astrocytoma is as significant as the distinction between WHO grade 1 and WHO grade 4 gliomas, and this scenario is not uncommonly encountered in clinical practice [6].

The emphasis on the well circumscription of pilocytic astrocytoma in the literature makes some pathologists uncomfortable making this diagnosis when infiltration is demonstrated histologically, especially if molecular tests are unavailable or cannot be performed as the situation in most laboratories around the world. This study aims to determine the infiltration growth of pilocytic astrocytoma in different brain locations.

## Materials and methods

Ethical approval from the institution's ethics committee was obtained. All cases diagnosed as "pilocytic astrocytoma" were retrieved from the pathology archives from 2008 to 2021. The pathological report was reviewed to obtain the clinical information (age, sex, location of the tumor), pathological description, and immunostains performed. In addition, the available pathological slides stained with hematoxylin and eosin (HE) and other immunostains were retrieved and examined for the following features: diagnosis, infiltrative vs. circumscribed tumor, and immunostaining characteristics.

The histological diagnosis was confirmed by applying the 2021 CNS WHO essential diagnostic criteria of pilocytic astrocytoma. These criteria were based on classic histological features of biphasic tumors containing both compact and microcystic areas. The molecular confirmation of mitogen-activated protein kinase (MAPK) activation was only required in cases with a glioma containing only piloid astrocytic neoplasm [1].

As the diagnosis was based mainly on histological features, the availability of immunostains was variable among cases. However, the following immunostains were available in some of the cases: anti-gliofibrillary acidic protein (GFAP), clone EP672Y, ready-to-use (RTU), anti-neurofilament protein ([NFP] 2F11, RTU), anti-Neu N (MAB377, dilution 1:400), anti-synaptophysin (MRQ-40, RTU), and anti-Ki67 (30-9, RTU). Immunostaining was performed on an automated BenchMark ULTRA Stainer from Ventana. Sections were cut at 4 µm thickness, and the quality of the immunostains was checked against positive and negative controls.

**Figure 1:**
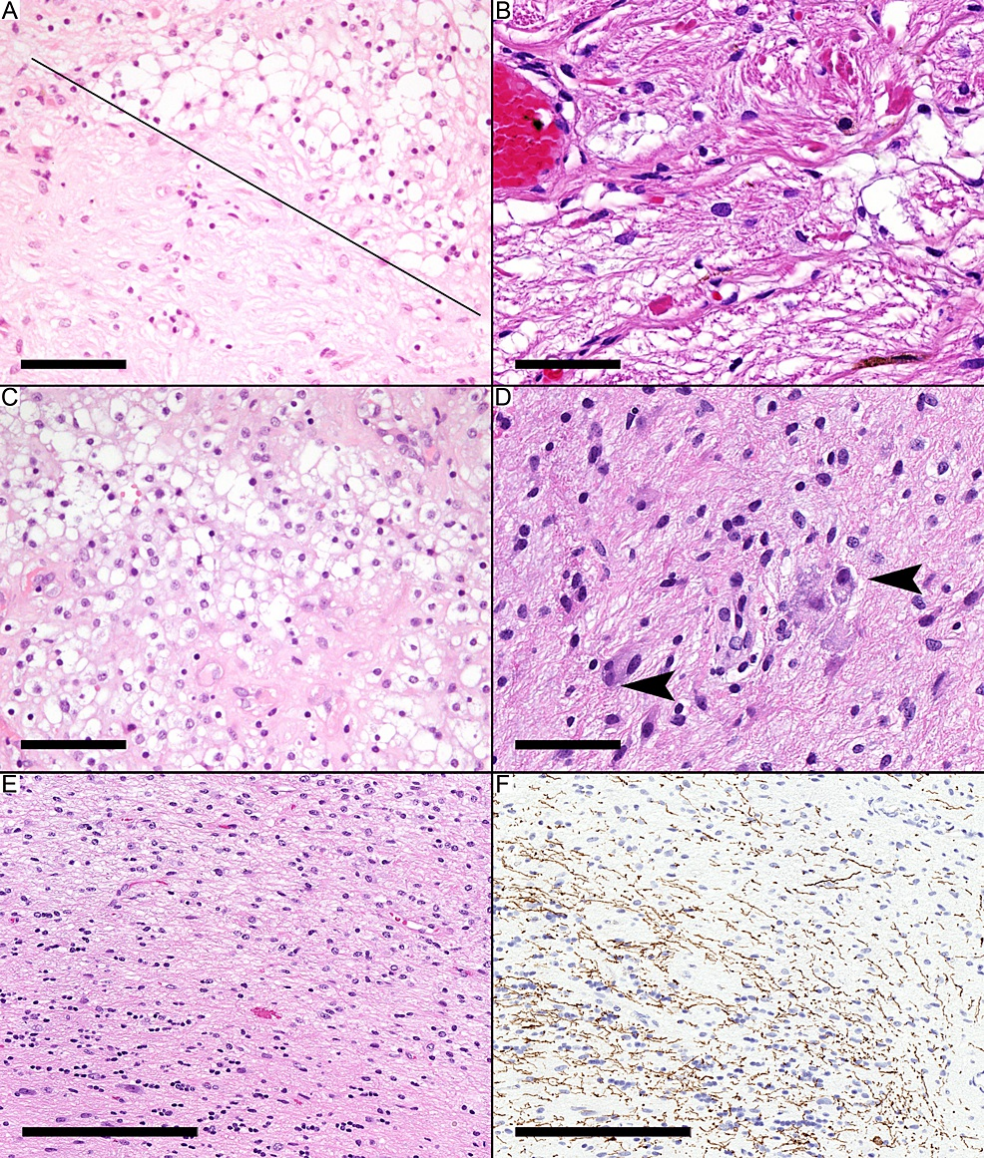
Morphological patterns and infiltration of a pilocytic astrocytoma (A) Pilocytic astrocytoma with the biphasic pattern (microcystic above the oblique line and fibrillary below the line). (B) Predominant fibrillary (bipolar astrocytes) pattern. (C) Predominant oligodendrocyte-like pattern. (D) Infiltration in the form of entrapped neurons (arrowheads). (E) Infiltration into adjacent brain tissue. (F) Neurofilament protein immunostaining highlights the infiltration into adjacent brain tissue. Scale bars: 60 µm (A-D), 200 µm (E, F).

**Figure 2:**
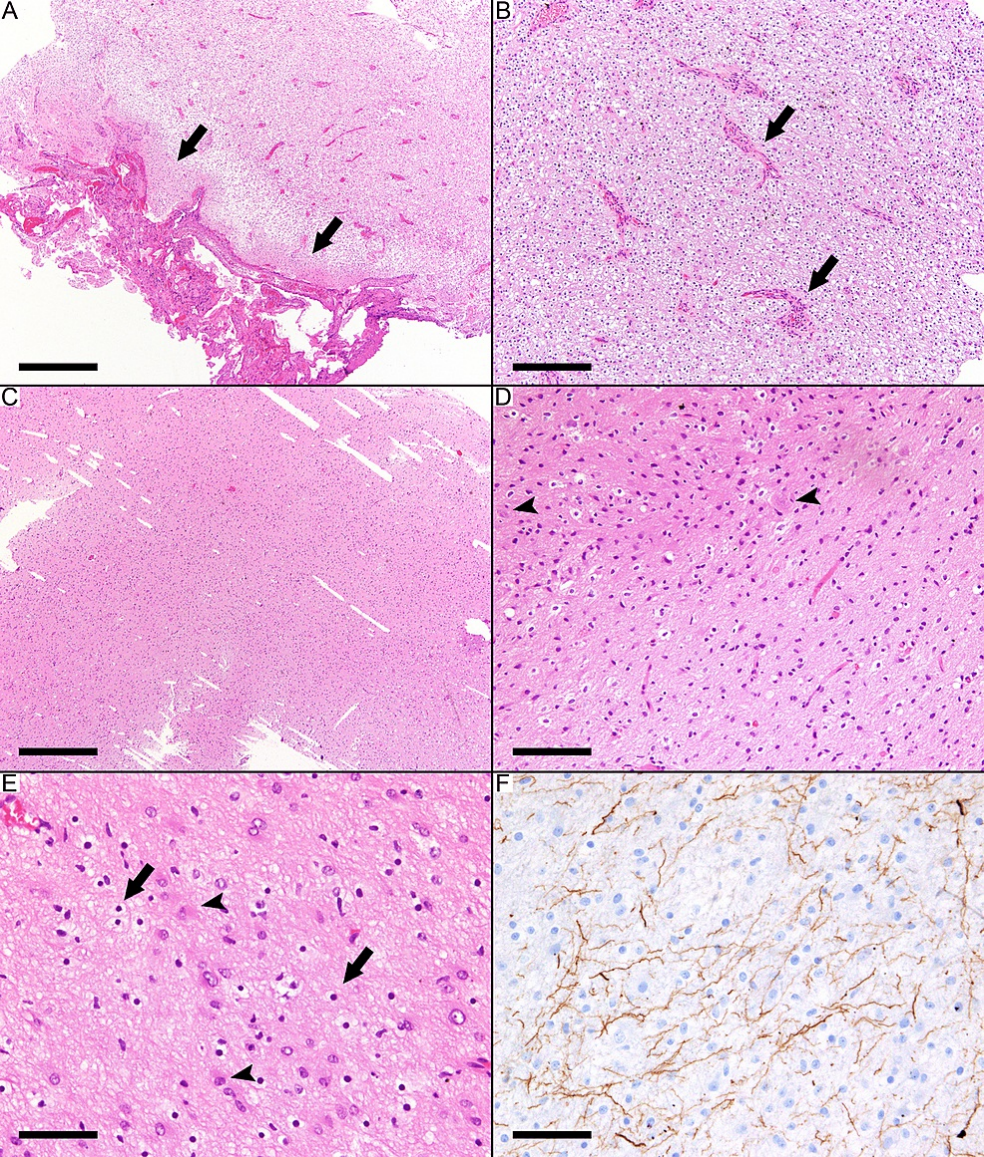
Brainstem pilocytic astrocytoma with infiltration (case 13) (A) Biphasic pattern with both oligodendrocyte-like area and fibrillary area (arrows) with bipolar astrocytes. (B) The oligodendrocyte-like area shows endothelial proliferation (arrows). (C-F) Diffuse infiltration with neoplastic cells infiltrating between the neurons and brain parenchyma. (D) Entrapped neurons (arrowheads) between the neoplastic cells. (E) Oligodendrocytes (arrows) and reactive astrocytes (arrowheads) among the neoplastic cells. (F) Neurofilament protein immunostaining shows many axons between the neoplastic cells, highlighting the infiltrative nature of the tumor. Scale bars: 500 µm (A, C), 200 µm (B), 100 µm (D), 60 µm (E, F).

**Figure 3:**
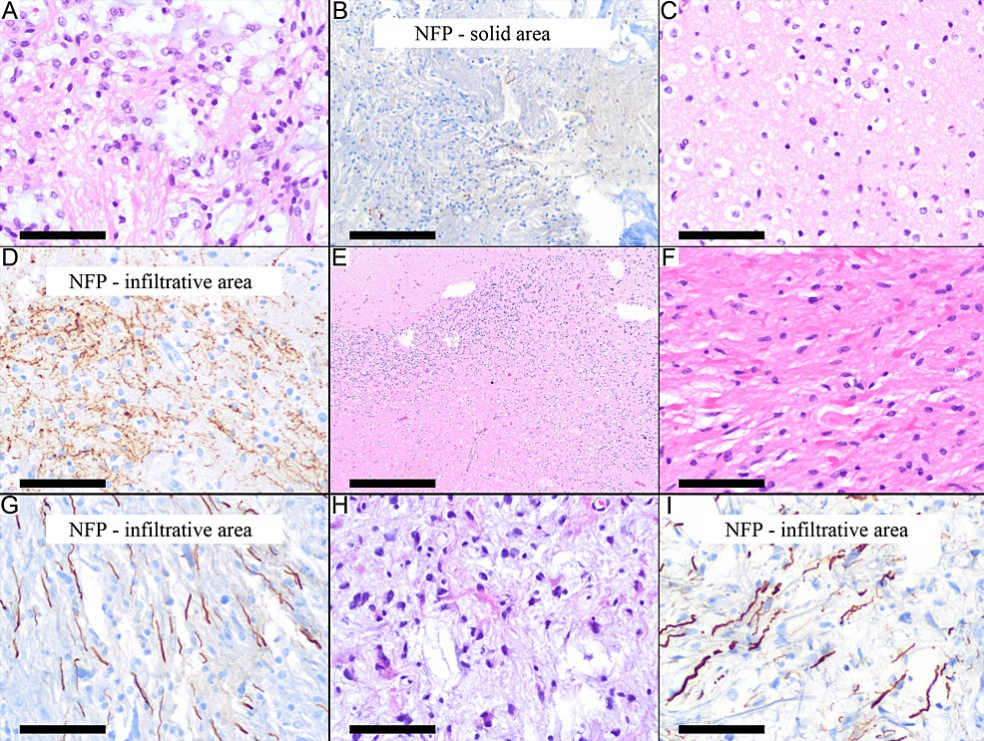
Pilocytic astrocytoma with infiltration in different sites Supratentorial (parietal lobe) pilocytic astrocytoma (Case 26) shows both a solid area (A, B) and an infiltrative area (C, D). (A) The solid area shows the biphasic pattern. (B) NFP immunostaining shows rare axons in this solid area. (C) Infiltrative focus with oligodendrocyte-like neoplastic cells among reactive astrocytes and oligodendrocytes. (D) NFP immunostaining highlights the infiltrative nature of this focus. (E) Cerebellar pilocytic astrocytoma (case 9) with neoplastic cell infiltration between the granular cell layer of the cerebellar cortex (right side of the figure). (F, G) The fibrillary area in the brainstem glioma (case 13) shows diffuse infiltration, as highlighted by many NFP-immunostained axons between the neoplastic cells. (H, I) Spinal cord pilocytic astrocytoma (case 20) shows neoplastic cells in a myxoid background among many NFP-immunostained axons. Scale bars: 100 µm (A, C, D, F-I), 200 µm (B), 500 µm (E). NFP, neurofilament protein

The morphological patterns of pilocytic astrocytoma were classified into three patterns according to the most recent WHO classification of CNS tumors [1]: (1) biphasic pattern containing both compact areas with bipolar astrocytes and Rosenthal fibers alternating with microcystic areas containing oligodendrocyte-like cells (Figure 1A), (2) predominantly compact areas with bipolar astrocytes (Figure 1B), and (3) predominantly dispersed with oligodendrocyte-like cells (Figure 1C). The term “well circumscribed" is defined here as a sharp demarcation between the neoplasm and the brain tissue. The term "infiltrative" is defined as either the presence of neurons among the neoplastic cells (Figure 1D) or the presence of neoplastic cells among normal brain parenchyma other than neurons (Figures 1E, 1F). The subarachnoid spread was not considered a form of infiltration.

## Results

The pathology reports of 48 cases were selected from the archive (2008-2021). Nine cases were excluded because no slides were available for review. The cohort included 20 males and 19 females (Table 1). The age ranged from 17 months to 31 years, with a mean of 10.8 years and a median of 8 years. The diagnosis of pilocytic astrocytoma was confirmed in 38/39 cases, and in one case, the diagnosis changed to dysembryoplastic neuroepithelial tumor (DNT). The morphological patterns were biphasic in 27 cases (pattern 1), predominantly bipolar fibrillary astrocytes in seven cases (pattern 2), and predominantly oligodendrocyte-like patterns in four cases (pattern 3).

**Table 1:** Clinical and pathological findings F, female; M, male; NA, not available/applicable; PA, pilocytic astrocytoma; Y, years

No.	Age	Sex	Location	Diagnosis	Pattern	Infiltration
1	1.5 Y	M	Cerebellum	PA	2	Yes
2	2 Y	M	Cerebellum	PA	1	Yes
3	2 Y	F	Cerebellum	PA	1	No
4	3 Y	M	Cerebellum	PA	2	Yes
5	4 Y	F	Cerebellum	PA	1	No
6	4 Y	F	Cerebellum	PA	1	Yes
7	4 Y	F	NA	PA	2	Yes
8	4 Y	F	Cerebellum	PA	1	Yes
9	4 Y	M	Cerebellum	PA	1	Yes
10	4 Y	M	Cerebellum	PA	2	No
11	5 Y	M	Frontal lobe	PA	2	No
12	5 Y	M	Parietal lobe	PA	1	No
13	5 Y	M	Brainstem	PA	1	Yes
14	6 Y	F	Cerebellum	PA	1	No
15	6 Y	M	NA	PA	1	No
16	6 Y	M	Cerebellum	PA	1	Yes, with entrapped neurons
17	6 Y	F	Cerebellum	PA	1	No
18	7 Y	F	Cerebellum	PA	3	No
19	7 Y	F	Parietal lobe	PA	1	Yes, with entrapped neurons
20	8 Y	M	Spinal T10-L1	PA	1	Yes
21	8 Y	F	Thalamus	PA	1	No
22	10 Y	F	Cerebellum	PA	1	Yes
23	11 Y	F	Cerebellum	PA	1	No
24	12 Y	F	Cerebellum	PA	2	Yes
25	12 Y	F	Frontal lobe	PA	1	No
26	13 Y	M	Parietal lobe	PA	1	Yes, with entrapped neurons
27	13 Y	F	Cerebellum	PA	1	Yes
28	15 Y	M	Cerebellum	PA	2	Yes
29	15 Y	M	NA	PA	3	No
30	16 Y	M	Occipital lobe	DNT	NA	NA
31	18 Y	M	Cerebellum	PA	1	No
32	18 Y	F	Cerebellum	PA	3	No
33	19 Y	F	Parietal lobe	PA	1	Yes, with entrapped neurons
34	19 Y	M	NA	PA	1	No
35	19 Y	F	Fronto-temporal	PA	1	No
36	23 Y	M	Suprasellar	PA	3	No
37	27 Y	M	Cerebellum	PA	1	Yes
38	29 Y	F	Cerebellum	PA	1	No
39	31 Y	M	Cerebellum	PA	1	No

The location of the pilocytic astrocytoma was cerebellar in 24 cases, supratentorial in seven cases, brainstem in one case, spinal cord in one case, thalamus in one case, and not documented in four cases. All cases showed well-circumscribed areas, while infiltration was present in 18/38 cases. In all cases, the infiltration was in the form of the presence of tumor cells among the nonneoplastic brain parenchyma. In four cases, entrapped neurons were present in the infiltrative foci. The infiltration was not restricted to cerebellar pilocytic astrocytoma (12/24, 50%) but was also present in 3/7 supratentorial, single brainstem, and single spinal cord cases. Brain infiltration was not discussed in the microscopic and pathological report diagnosis sections in all cases except for the brainstem case (case 13).

Brainstem pilocytic astrocytoma (case 13) is an example of a case with the typical features of pilocytic astrocytoma and areas of diffuse infiltration. The neoplasm showed a biphasic pattern with both oligodendrocyte-like and fibrillary piloid areas (Figure 2A). Endothelial proliferation is present in the oligodendrocyte-like area (Figure 2B). Diffuse infiltration with neoplastic cells infiltrating between the neurons and brain parenchyma was observed by HE staining (Figures 2C-2E). The infiltrative nature was highlighted by the NFP immunostaining, which stains the axons between the neoplastic cells (Figure 2F).

Infiltration in pilocytic astrocytoma was identified in different CNS locations, including the supratentorial (Figures 3A-3D), cerebellum (Figure 3E), brainstem (Figures 3F, 3G), and spinal cord (Figures 3H, 3I).

## Discussion

Pilocytic astrocytomas are common low-grade gliomas that account for 5% of CNS primary tumors [7] and have a predilection to affect children. Histologically show typical biphasic components with compact fibrillary piloid and oligodendrocyte-like microcystic areas. They may show microvascular glomeruloid proliferation and small necrosis but typically low proliferative activity [8]. Cases with brisk mitotic activity are considered anaplastic pilocytic astrocytoma [9], and the morphology of these cases overlaps with anaplastic astrocytoma with piloid features [10].

All cases showed molecular evidence of MAPK pathway activation [11-13]. The most common alteration is BRAF duplication and fusion with KIAA1549 [14,15]. In addition, they commonly show immunopositivity for GFAP [16], S100, OLIG2, and, in some cases, synaptophysin [1].

They are well-known as well-circumscribed tumors both radiologically and histologically. A thorough and specific search of histological infiltration in pilocytic astrocytoma, including cerebellar, supratentorial, brainstem, and spinal examples, will result in findings of infiltrative areas in a significant number of examined cases (18/38, 47% in this study). The infiltrative foci can be overlooked or ignored, as the pathological reports of all cases in the current study did not describe these foci except for the brainstem case. In that case, the recognition of the infiltration made the pathologist comment on the significance of this infiltration and entertain the possibility of “diffuse midline glioma” in the differential diagnosis. However, the latter diagnosis of pilocytic astrocytoma was preferred with the typical morphological "solid" areas.

The well circumscription of pilocytic astrocytoma is emphasized in the literature, and, usually, it is the first tumor to be discussed under the category of “circumscribed astrocytic gliomas” [1,17]. The descriptive term “circumscribed" is not well defined, but, generally, it is used to describe a distinct demarcation of the tumor from the adjacent brain parenchyma with no evidence of histological infiltration. Infiltration in the gray matter can be recognized by the neoplastic cells between the neurons, and infiltration in the white matter can be recognized by the presence of neoplastic cells between the nonneoplastic oligodendrocytes. NFP immunostaining highlights infiltration as it stains prominent axons in the white matter [17].

Early pathological studies of pilocytic astrocytoma described cases with histological infiltration despite the well-demarcated appearance radiologically. Coakley et al. found that 64% of 56 pilocytic astrocytomas infiltrated the adjacent parenchyma [3]. Hayostek et al. found that 15/105 cerebellar pilocytic astrocytoma were of the diffuse type [2]. However, the diffuse nature of these tumors did not have statistical significance in survival [2]. The latest WHO classification of CNS tumors described pilocytic astrocytoma as circumscribed astrocytic gliomas. However, the microscopic description indicated that some pilocytic astrocytoma might resemble diffuse astrocytoma because of the "surprising degree of infiltration," and others may resemble ganglioglioma because of the presence of entrapped neurons [1].

The diagnosis of a histologically non-infiltrative biphasic tumor as pilocytic astrocytoma is straightforward, but infiltrative examples may pose a challenge to differentiate it from diffuse astrocytoma. The differential diagnosis of the well-circumscribed tumors is primarily other low-grade tumors, such as ganglioglioma, rosette-forming glioneuronal tumor, and pilomyxoid astrocytoma. On the other hand, the differential diagnosis of the infiltrative tumors will include high-grade astrocytoma. In this example, the distinction between these two entities significantly impacts the treatment and prognosis and is a very challenging situation [13]. The presence of necrosis and glomeruloid microvascular proliferation in such tumors is acceptable as CNS WHO grade 1 in pilocytic astrocytoma, while they upgrade the tumor to CNS WHO grade 4 if diagnosed as infiltrative astrocytoma.

The recent advances in molecular pathology introduced more complicated situations, mainly when "selected" molecular tests were only performed. Here are a few examples of these possible situations when an astrocytic tumor with infiltration is encountered: First, IDH wild type in “adult-type” astrocytoma may indicate glioblastoma (WHO grade 4) if “diffuse” astrocytoma was the histological diagnosis, while in pilocytic astrocytoma it is the expected IDH status. Second, H3 p. K28 M (K27 M) in midline tumors is more complicated, as it indicates CNS WHO grade 4 in infiltrative astrocytoma [5] but does not change the grade in pilocytic astrocytoma. The emphasis on the distinction between these two tumors was based on the typical circumscription of midline pilocytic astrocytoma [5]. Therefore, it will be very challenging to interpret the significance of this mutation in pilocytic astrocytoma with infiltration, as these cases will fulfill the diagnostic criteria for midline glioma, H3 K27-altered [5].

In this study, 18/38 cases showed infiltrative foci that could be used as a supportive feature of infiltrative glioma. However, all pilocytic astrocytoma in this study, including the 18 cases with infiltrative foci, showed areas of compact non-infiltrative glioma typical of pilocytic astrocytoma. The presence of the typical biphasic pattern of pilocytic astrocytoma (27/38 cases in this study) is not documented, up to my knowledge, in infiltrative astrocytoma. As such, their presence can be used to distinguish between "diffuse"/infiltrative glioma and pilocytic astrocytoma with infiltration.

The results of molecular studies, when available, can be helpful but also have limitations. The detection of KIAA 1549-BRAF duplication and fusion is the most expected alteration (70-80%) in pilocytic astrocytoma but is also described in 5-10% diffuse astrocytoma [18]. The diagnosis of pilocytic astrocytoma is mainly based on morphological assessment, and thus it is essential to appreciate all the possible pitfalls.

The limitation of this study is the retrospective nature of the study and the lack of molecular tests on these cases. However, the diagnosis of pilocytic astrocytoma depends on morphological assessment, and the lack of molecular alteration should not affect the diagnosis, but it could add additional pathological-molecular correlation data.

## Conclusions

This study confirmed the presence of histological infiltrative foci in pilocytic astrocytoma, as it was present in 18/38 cases in this study. Furthermore, this study determined that in cases with typical morphological features of pilocytic astrocytoma, areas showing brain infiltration are acceptable and should not be used as an exclusion criterion of the diagnosis or affect the pathologist's certainty about the diagnosis.

The interpretations of the recent molecular markers, such as IDH status and H3 K27M, into the final integrated diagnosis, depend primarily on the morphological diagnosis (pilocytic astrocytoma vs. diffuse astrocytoma). The impact of uncertainty of the diagnosis in these cases will introduce CNS WHO grade 4 tumors in the differential diagnosis, which have different treatments and prognoses.
